# Supplementary material for: Factors Affecting Physical Activity in People with Dementia: A Systematic Review and Narrative Synthesis
Source: Behav Sci (Basel). 2023 Nov 8;13(11):913. doi: 10.3390/bs13110913 (PMC10669736; doi:10.3390/bs13110913)
Supplement: Supplementary file 1 [file behavsci-13-00913-s001.zip › Table S1_Supplementary material_Factors affecting physical activity in people with dementia_Revised Nov 7 proofreading confirmed.pdf]

Table S1. Data extraction table for included studies: success of implementation, barriers, facilitators, motivators and recommendations given by authors.

Reference	Successful number[a]	Successful author[b]	Barriers	Facilitators	Motivators[c]	Recommendations
Barrado-Martín, Heward, Polman and Nyman [54]	54% (0-179%)[d]	NA	• Not enjoying the PA<br>• Competing commitments<br>• Health issues<br>• Occasional forgetfulness<br>• Busy day<br>• Holiday/traveling<br>• Dyads not living together	• Dyadic approach<br>• Reminders around the house<br>• Tailoring exercises to participant's needs and environment<br>• Possibility to carry booklet to somewhere else<br>• Development of habit/daily routine<br>• Feeling that any difficulty could be clarified by instructor	• Expectations of getting better/achieving benefits<br>• Feeling committed<br>• Doing it together with caregiver<br>• Enjoyment of PA	• More visual material to support home PA<br>• Developing exercise routines
Barrado-Martín, Heward, Polman and Nyman [55]	75-83%[e]	Yes	• Amount of content delivered<br>• Lack of guidance at home<br>• Negative perception of Tai Chi<br>• Low sense of efficacy for caregiver or PwD	• Dyadic approach/support from caregiver<br>• Quality instructor<br>• Memory aids<br>• Adaptation to participants needs<br>• Positive environment<br>• Positive feedback	• Perceived benefits by both PwD and caregiver<br>• Social engagement	• Dyadic approach<br>• Combination of class and home practice<br>• Adapted instructional methods<br>• Individual approach<br>• Guidance with home PA<br>• Explain potential benefits
Dawson, Judge and Gerhart [53]	99.0%[d]	Yes	• Frustration, inability to complete PA<br>• Poor adherence despite willingness<br>• Boredom	• Keep it short and simple<br>• External memory aids<br>• Learning by modelling<br>• PwD chooses activity<br>• Using familiar activities		• Strength-based approach is a useful framework for guiding implementation
Donkers, van der Veen, Vernooij-Dassen, Nijhuis-van der Sanden and Graff [50]	NA	Yes	• Collaboration between healthcare and welfare professionals<br>• Difficulty transferring dyads to a professional involved in delivering the PA	• Focused on social participation<br>• Community-based<br>• Improved relationship with professionals<br>• Low-cost opportunity for PA	• Experience of improvements in dyads' functioning and social participation	• Train professionals involved in intervention delivery (care planning, shared decision making, helping dyads set priorities)

			• PwDs already received PA treatment<br>• Limited organised social activities<br>• Difficulty finding appropriate social activities (insurance)<br>• Lack of motivation by PwD<br>• Caregivers perceived high burden			
Hancox, Van Der Wardt, Pollock, Booth, Vedhara and Harwood [56]	25% high adherence 40% low adherence[f]	NA	• Below a threshold of cognitive capacity<br>• Low practical/emotional support<br>• Lack purpose	• Practical and emotional support from professionals and caregivers<br>• Memory support<br>• Exercise routine	• Believe in and experience of benefits<br>• Positive past experience of sport<br>• Having a meaningful purpose	• Develop an individual exercise routine<br>• Strong emotional support from caregivers<br>• Explain purpose of exercises
Henskens, Nauta, Scherder, Oosterveld and Vrijkotte [58]	72% completed follow-up	Yes	• Understaffing<br>• Limited time available for personalized care and stimulation<br>• Doubts about at which stimulation becomes a burden	• Individually based instruction<br>• Adapted to physical capabilities and personal preferences<br>• Support from nursing staff and caregivers<br>• Trained nursing staff<br>• Nursing staff prepared to deliver PA	• Majority experienced benefits<br>• Staff experienced benefits of PA	• Take understaffing and limited time into consideration
Henwood, Neville, Baguley and Beattie [59]	63.2%[d]	NA	• Facility resources: under-resourcing and under-staffing<br>• Engagement	• Invested staff		• Care providers need to embrace the opportunity and support staff and residents
Kruse, Cordes, Schulz and Wollesen [60]	72% (53-100%)[d]	Yes	• Armrests	• Training modified to requirements PwDs<br>• Instruction methods<br>• Adaptation of exercises		• Consult nurse in advance<br>• Individualized instructions combining verbal, auditory, visual and tactile cues were most effective

				• Individual instructions/supervision<br>• One-on-one support<br>• Activities related to everyday life<br>• Support from relatives		• One-on-one support<br>• Limited group size<br>• Exercises more task-oriented and related to ADL<br>• Tailored to PwD's capacities
			•			•
				•		
MacAndrew, Kolanowski, Fielding, Kerr, McMaster, Wyles and Beattie [57]	86%[d]	Yes	• Weather<br>• Strict tailored timing of walk<br>• PwD fatigue		• Benefits for PwDs and staff<br>• Perceived having benefits for PwD's<br>• Enjoying walk neighbourhood	• Shortened duration of walk<br>• Walks in de afternoon<br>• Group walks for socialisation<br>• Support (professional, caregiver) for walks
Sondell, Rosendahl, Gustafson, Lindelöf and Littbrand [51]	73.4%[d]	Yes	• Low motivation<br>• Health issues<br>• Tiredness<br>• Fear	• Functional exercises easy to follow<br>• Exercises individualized		• Additional weekly session<br>• Individualization of session times to daily routines.<br>• Strategies to enhance motivation
Tak, van Uffelen, Paw, van Mechelen and Hopman-Rock [52]	66% completed follow-up<br>53%[d]	NA	• Practical reasons (time and location)<br>• Physical limitations<br>• Dissatisfaction with content (intensity) program<br>• Adjust exercise intensity<br>• Cost	• Trained instructors<br>• Quality aspects intervention (instructor, content)	• Clear benefits reported by PwDs	• Adjust intensity of exercise program<br>• Providing high-quality instructor and feasible content<br>• Address specific outcome expectations
Teri, Logsdon, McCurry, Pike and McGough [61]	81% completed 1st follow-up; 55% 2nd follow-up<br>91%[g]	Yes	• Identifying appropriate participants<br>• Understanding protocol<br>• Adhering to time schedule	• Individualized exercises<br>• Educating caregivers<br>• Behavioural problem solving<br>• Support for AAA agencies	• Experienced benefits by dyads	• Adequate financial resources<br>• Staff must be trained, supervised and maintained
Yu and Kolanowski [62]	95.8 & 100%[d]	Yes	• Problems with attention and memory	• Training trainers for supervision of exercise		• Monitoring strategies<br>• Regular exercise programs

	• Burden on caregivers<br>• Caregivers doubts about potential benefits<br>• Safety<br>• Loss of motivation/resources<br>• Disruptive behaviour during activity<br>• Lack of exercise programs for PwDs<br>• Obtaining collaboration form care practitioner	• Trusting relationship between trainer, PwD and caregiver<br>• Caregiver collaboration<br>• Educating caregivers and participants<br>• Networking<br>• Providing transportation		• Trusting relationship training, PwD and caregiver

[a] Objective measure of success: adherence/attendance rates

[b] Subjective measure of success: conclusion of the author

[c] Motivators were classified by the author (W.R.F)

[d] Treatment adherence (% of recommended PA completed/ % attended sessions/attendance rate)

[e] Average attendance to classes

[f] Low = < 3 times a week, high = > 5 times a week

[g] Dyads engaged in assigned homework

Abbreviations: AAAs = Area Agencies on Aging; PwD = people with dementia; NA = not available/not announced; PA = Physical activity.
