# Supplementary material for: Factors Affecting Physical Activity in People with Dementia: A Systematic Review and Narrative Synthesis
Source: Behav Sci (Basel). 2023 Nov 8;13(11):913. doi: 10.3390/bs13110913 (PMC10669736; doi:10.3390/bs13110913)
Supplement: Supplementary file 1 [file behavsci-13-00913-s001.zip › Table S2_Supplementary material_Factors affecting physical activity in people with dementia Revised Nov 7 Proofreading confirmed.pdf]

Table S2. Factors influencing PA participation in people with dementia based on empirical implementations with new factors printed in bold.

Barriers	Motivators[a]	Facilitators
	**Intrapersonal level**	
	Physical or Mental Health	
• Health conditions [51,52,54]<br>• Problems with cognition; attention, memory and confusion [54,56,62]<br>• Lack of motivation [50,51,62]<br>• Fatigue [51,57]<br>• **Low sense of efficacy PwD (or caregiver**) [55]<br>• **Lack of confidence at home [55]**<br>• Emotional barriers (fear) [51]<br>• Disruptive behaviour PwD [62]	• Perceived/experienced physical benefits [51,52,55-58,61]<br>• Expectation/believe in possible physical benefits [54,56]<br>• Meaningful purpose of PA [56]	• Adapt exercises to physical capabilities/patients' needs [51,55,57,58,60,61]<br>• Strategies to overcome memory problems (external memory aids) [53,55,56]<br>• **Behavioural problem solving [61]**
	Individual preferences	
• **Negative perception/dislike of specific PA [52,55]**<br>• **Not enjoying PA [54]**<br>• **Boredom or lack of enthusiasm [53]**<br>• **Lack of accommodating PA [52]**<br>• **Difficulty finding appropriate activities [50]**	• Enjoyment of PA [54,57]<br>• Positive (past) association with PA [56]<br>• Sense of commitment [54]<br>• **Using preferred or familiar activities/hobbies [53,58]**<br>• **PwD chooses PA [53]**<br>• **Activities related to everyday life [60]**	
	**Interpersonal level**	
	General support from informal caregiver, family or health professional	
• Caregiver factors (burden, doubts) [50,62]<br>• **Caregiver and PwD not living together [54]**<br>• **Lack of practical and emotional support [56]**<br>• **Lack of guidance [55]**<br>• Concerns regarding safety [62]	• **Doing it together with caregiver [54]**	• Caregiver/family support/ dyadic approach [54-56,60,62]<br>• Advice, feedback and support experts [50,54,56,58]<br>• **Quality/trained of instructor/staff [52,55,58,62]**<br>• **Trusting relationship trainer, PwD and caregiver [50,62]**<br>• Educating caregivers and PwDs [61,62]<br>• **Positive feedback/environment [55]**
	Support from staff in nursing homes	

• **Understaffing [58,59]**<br>• **Limited time for personalized care and stimulation [58]**<br>• **Staff doubts [58]**		• **Benefits for staff [57,58]**<br>• **Staff prepared to deliver PA [58,59]**
	Social identification	
	• Social participation/connectedness [50,55]<br>• Networking [62]	
	**Community level**	
	Structural and organizational (intervention) factors	
• Limited organised activities [50,62]<br>• Competing commitments (activities, holiday,) [54]<br>• **Amount of content delivered [55]**<br>• **Strict timing of walks [57]**<br>• **Armrests of chairs [60]**<br>• Practical reasons (time, location) [52]<br>• **Cost [52]**<br>• **Collaboration between experts [50]**<br>• **Difficulty transferring dyads to expert [50]**<br>• Collaboration practitioner [62]<br>• **Understanding protocol [61]**<br>• **Adhering to time schedule [61]**		• **Instruction methods [53,58,60]**<br>• **Program/intervention accommodation [60]**<br>• Development of habit/daily routine [54,56]<br>• Program characteristics [51,52]<br>• **Individual supervision [60]**<br>• Providing transportation [62]<br>• **Low cost PA opportunity [50]**<br>• **Support AAA agencies [61]**
	Physical environment	
• Environment (weather, inaccessible, not safe) [57]		

[a] Motivators were classified by the author (W.R.F)

New barriers, facilitators and motivators are presented in bold. Abbreviations: AAAs = Area Agencies on Aging; PwD = people with dementia.
